# Comparison of Yizhiqingxin formula extraction methods and their pharmacodynamic differences

**DOI:** 10.3389/fnins.2023.1097859

**Published:** 2023-02-16

**Authors:** Wei Wei, Hui Pei, Li-Na Ma, Rui Zheng, Qiao-Yi Huang, Su-rui Chang, Yu Cao, Hao Li

**Affiliations:** ^1^Xiyuan Hospital, China Academy of Chinese Medical Sciences, Beijing, China; ^2^Wangjing Hospital, China Academy of Chinese Medical Sciences, Beijing, China

**Keywords:** Yizhiqingxin formula (YQF), Alzheimer’s disease, mice, Chinese medicine, pharmaceutical technology

## Abstract

**Objectives:**

This study compared different extraction methods of Yizhiqingxin formula (YQF) and its neuroprotective effects based on pharmacodynamic indices such as learning and memory ability, brain tissue histopathology and morphology, and inflammatory factor expression in a mouse model of Alzheimer’s disease (AD).

**Methods:**

The pharmaceutical components of YQF were extracted using three extraction processes, and the components were analyzed by high performance liquid chromatography. Donepezil hydrochloride was used as a positive control drug. Fifty 7–8-month-old 3 × Tg AD mice were randomly divided into three YQF groups (YQF-1, YQF-2, and YQF-3), a donepezil group, and a model group. Ten age-matched C57/BL6 mice were used as normal controls. YQF and Donepezil were administered by gavage at a clinically equivalent dose of 2.6 and 1.3 mg⋅kg^–1^⋅d^–1^, respectively, with a gavage volume of 0.1 ml/10 g. Control and model groups received equal volumes of distilled water by gavage. After 2 months, the efficacy was evaluated using behavioral experiments, histopathology, immunohistochemistry, and serum assays.

**Results:**

The main components in YQF are ginsenoside Re, ginsenoside Rg1, ginsenoside Rb1, epiberberine, coptisine chloride, palmatine, berberine, and ferulic acid. YQF-3 (alcohol extraction) has the highest content of active compounds, followed by YQF-2 (water extraction and alcohol precipitation method). Compared to the model group, the three YQF groups showed alleviated histopathological changes and improved spatial learning and memory, with the effect in YQF-2 being the most significant. YQF showed protection of hippocampal neurons, most significantly in the YQF-1 group. YQF significantly reduced Aβ pathology and tau hyperphosphorylation, decreased expressions of serum pro-inflammatory factors interleukin-2 and interleukin-6 as well as serum chemokines MCP-1 and MIG.

**Conclusion:**

YQF prepared by three different processes showed differences in pharmacodynamics in an AD mouse model. YQF-2 was significantly better than the other extraction processes in improving memory.

## 1. Introduction

Alzheimer’s disease (AD), a type of dementia, is a common clinical neurodegenerative disease in the elderly that seriously affects learning and memory functions as well as daily behavioral abilities and brings a heavy burden to families and society. Studies on AD have shown a plethora of genes that can be grouped into a polygenic risk score (Scheltens et al., 2021); however, there is a lack of effective therapeutic drugs that are known to treat AD. Therefore, traditional Chinese medicine (TCM) compounds and their extracts are important in the prevention and treatment of AD with multichannel and multitarget therapeutic characteristics (Pei et al., 2020). The Yizhiqingxin formula (YQF) is based on the TCM theory of dementia “deficiency, stasis, and toxicity,” and is composed of *Panax ginseng* C.A.Mey (Renshen), *Coptis chinensis* Franch (Huanglian), and *Ligusticum chuanxiong* S.H.Qiu, Y.Q.Zeng, K.Y.Pan, Y.C.Tang and J.M.Xu (Chuanxiong) in a ratio of 9:5:6. It can significantly improve spatial learning and memory capacity in AD model mice, delay the pathological processes of AD, and exert therapeutic effects by regulating oxidative stress and neuroinflammation, according to basic research (Yang et al., 2019; Ma et al., 2020). It has good potential as a novel drug. To further expand the clinical applications of YQF and develop a safe and effective TCM compound, we designed three compound extraction processes combining the physicochemical characteristics of the medicinal components in the compound, and we selected donepezil hydrochloride, which has definite efficacy in AD, as a control drug for observation. The efficacy of YQF prepared by three different extraction processes was evaluated in the AD mouse model using behavioral and pathological efficacy indexes to evaluate the extraction processes and provide an experimental basis for future studies.

## 2. Materials and methods

### 2.1. Animals

In this study, 50 male 3 × Tg AD mice (7–8 months) were used as model mice (No: 14002A), with 10 male wild-type C57/BL6 mice as normal controls. All animals were purchased from Beijing HFK Bioscience Co., Ltd., (Beijing, China). Animal experiments were approved by the Ethics Committee of Xiyuan Hospital of the China Academy of Chinese Medical Sciences (No. 2021XLC032-2). All animals were housed in an specific-pathogen-free animal room at Xiyuan Hospital, Chinese Academy of Traditional Chinese Medicine at 22 ± 3°C with a 12 h/12 h light–dark cycle, 50% ± 10% relative humidity, and *ad libitum* water and food. Animals were acclimatized to their environment for 1 week before experiments.

### 2.2. Drugs, chemicals, and reagents

YQF consists of *Panax ginseng* C.A.Mey (Renshen), *Coptis chinensis* Franch (Huanglian), and *Ligusticum chuanxiong* S.H.Qiu, Y.Q.Zeng, K.Y.Pan, Y.C.Tang and J.M.Xu (Chuanxiong). These herbs were purchased from Hebei Baicao Kangshen Pharmaceutical Co., Ltd., (Hebei, China). Donepezil hydrochloride (production lot No. 2104113; Shenzhen Anlixin, Shenzhen, China) was purchased from Eisai (Tokyo, Japan). Ginsenosides Rg1 reference substance, purity 99.70% (A0237); ginsenosides Re reference substance, purity 99.52% (A0244); ginsenosides Rd reference substance, purity 98.55% (A0245); ginsenosides Rb1 reference substance, purity 98.58% (A0234); ferulic acid reference substance, purity 99.32% (A0050); provided by Chengdu Desite Biotechnology Co., Ltd., Chengdu, China. Ginsenosides Rh2 reference substance, purity 98% (B21729); Ginsenosides Rg2 reference substance, purity 98% (B21727); epiberberine reference substance, purity 98% (B20108); coptisine chloride reference substance, purity 98% (B21438); palmatine reference substance, purity 98% (B21646);berberine reference substance, purity 98% (B21379); and provided by Shanghai Yuanye Biotechnology Co., Ltd., Shanghai, China.

### 2.3. Instruments and equipment

The following instruments and equipment were used for experiments: Morris water maze (MWM; ZS-001; Beijing Zhongshidichuang Science and Technology Development Co., Ltd., Beijing, China); fluorescence microscope (Eclipse E100; Nikon, Tokyo, Japan); imaging system (DS-U3; Nikon); flow cytometer (NovoCyte D1040; Hangzhou Essen Pharmaceutical Research Co., Ltd., Hangzhou, China); QBplex^®^ EZPrep plate washer (VM1001; Beijing Kuangbo Biotechnology Co. Ltd., Beijing, China).

### 2.4. Yizhiqingxin formula extraction processes

YQF-1: raw herbs were firstly decocted for 1.5 h in water in an amount of eight times the weight of materials. Then filtered to get the residue and decocted again for 1 h with six times amount of water. Combined filtrates were concentrated to 384 ml (1.04 g/ml). YQF-2: raw herbs were firstly decocted for 1.5 h in water in an amount of eight times the weight of materials. Then filtered to get the residue and decocted again for 1 h with six times amount of water. Combined filtrates were concentrated to 400 ml and ethanol was added to make the alcohol content to 60% (632 ml). The solution was allowed to stand for 24 h and filtered. Then, ethanol was removed, and the total amount of solution was adjusted to 384 ml (1.04 g/ml). YQF-3: Reflux Extracted twice for 2 h each with 75% ethanol in the amount of six times the volume of raw drugs. Combined filtrates and removed ethanol. Then the solution was adjusted to 384 ml. All extracted solutions (1.04 g/ml) were stored at 4°C and equilibrated to room temperature before administration by gavage. After drying, we got YQF-1 powder 58.2 mg/ml, YQF-2 powder 71 mg/ml, and YQF-3 powder 112.2 mg/ml.

### 2.5. High-performance liquid chromatography (HPLC) analysis of YQF

The reference standards or YQF powder were precisely weighed and dissolved with methanol solution. Inertsil C18 (150 mm × 4.6 mm, 5 μm) was used as the stationary phase for the chromatographic separation. The compounds were identified by individual peak retention times compared to reference substances. The detection wavelength of the main components of Panax ginseng C.A.Mey (Renshen) in YQF was 203 nm, with a column temperature at 35°C. The flow rate was 1 ml/min and the total injection volume was 20 μL. The mobile phase consisted of solvent A (acetonitrile) and solvent B (water) with the following gradient elution: 18% A at 0–40 min; 21% A at 40–42 min; 26% A at 42–46 min; 32% A at 46–66 min; 33.5% A at 66–71 min; 38% A at 71–86 min; 65% A at 86–96 min; 85% A at 96–103 min (Guo, 2014). The detection wavelength of the main components of Coptis chinensis Franch (Huanglian) in YQF was 345 nm, with a column temperature at 25°C. The flow rate was 1 mL/min, and the total injection volume was 5 μL. The mobile phase consisted of 70% solvent A (0.05% trifluoroacetic acid) and 30% solvent B (acetonitrile). The detection wavelength of the main components of Ligusticum chuanxiong S.H.Qiu, Y.Q.Zeng, K.Y.Pan, Y.C.Tang and J.M.Xu (Chuanxiong) in YQF was 294 nm, with a column temperature at 30°C. The flow rate was 1 mL/min, and the total injection volume was 5 μL. The mobile phase consisted of 60% solvent A (0.05% trifluoroacetic acid) and 40% solvent B (methanol).

### 2.6. Drug administration

Mice in the YQF-1, YQF-2, and YQF-3 groups were administered clinically equivalent doses of 2.6 g⋅kg^–1^⋅d^–1^ (Yang et al., 2019), and donepezil mice were administered clinically equivalent doses of 1.3 mg⋅d^–1^. The control group and the model group received the same volumes of distilled water by gavage. Gavage was administered once a day for 2 months. Two animals in the model group, One animal in the YQF-2 group and two animals in the YQF-3 group died before the end of the experiments.

### 2.7. Sample collection and preparation

After behavioral experiments, blood and brain tissues were collected. Blood was placed in centrifuge tubes, left for more than 1 h at room temperature, centrifuged (3,000 rpm, 10 min) to collect the serum, and stored at −80°C for examination. Brains were removed, and sagittally cut into left and right hemispheres, fixed in 4% paraformaldehyde, embedded in paraffin, and sectioned.

### 2.8. Behavioral experiments

MWM was performed after the end of the gavage. The day before MWM testing, each experimental mouse was placed into the pool without a platform for 60 s to adapt to the environment. For training, spatial navigation experiments were performed for five consecutive days; each mouse was trained twice a day for 60 s each using different entry points. The mice were placed in the water facing the wall of the pool, and the time between entering the water and finding the escape platform (with all limbs on the platform for 6 s; escape latency) was recorded using a video tracking system. On day 6, the escape platform was removed for spatial exploration experiments; mice were placed in the water facing the wall of the pool (at the midpoint of the third quadrant), and the number of times the mouse crossed the original platform area within 60 s was recorded, the time in the target quadrant and distance moved in the target quadrant were also recorded to examine memory of the target quadrant where the original platform was located. A quiet environment with a stable light source and a water temperature of 23 ± 1°C were maintained during experiments.

### 2.9. Histopathological observation of brain tissue

Brain tissues were embedded in paraffin and sectioned. Then sections were processed as follows: Xylene for 20 min, two times; 100% ethanol for 5 min, two times; 75% ethanol for 5 min; and tap water rinsing. Sections were subjected to hematoxylin–eosin (HE) and toluidine blue (Nissl) staining. Hematoxylin–Eosin Staining Kit (G1003; Wuhan Servicebio Technology Co., Ltd., Wuhan, China) was used for HE staining, and Toluidine blue staining solution (G1032; Wuhan Servicebio Technology Co., Ltd.) was used for Nissl staining.

### 2.10. Multiplex immunoassays for flow cytometry

IL-2, IL-4, IL-6, IL-12p70, IL-17A, IP-10, MCP-1, MCP-3, IFNγ, RANTES, MIG, Eotaxin, and keratinocyte-derived chemokine (KC) were measured using Mouse Group 1 16-plex kit (C261116; Beijing QuantoBio Biotechnology Co., Ltd., Beijing, China) in this experiment. Briefly, 45 μl/well of serum sample and 45 μl/well of mixed capture beads were added in a 96-well plate, shaken and incubated in darkness for 60 min at room temperature, and washed in 100 μl/well washing buffer for three times. Then, 25 μl/well of biotin-conjugated antibodies was added, shaken and incubated in darkness for 30 min at room temperature, followed by three washings in 100 μl/well washing buffer. Next, 25 μl/well of streptavidin-PE was added, shaken and incubated in darkness for 20 min at room temperature. After three times of washing with 100 μl/well washing buffer, 150 μl/well of Reading buffer was added and the fluorescence signals were detected using flow cytometer (NovoCyte D1040; Hangzhou Essen Pharmaceutical Research Co., Ltd., Hangzhou, China). FCAP Array 3.0 software was used to analyze the levels of chemokines and inflammatory factors.

### 2.11. Immunohistochemistry

After deparaffinized and gradual hydration through graded alcohols, paraffin sections of brain tissue were immersed in EDTA antigen retrieval buffer (pH 6.0) and maintained at a sub-boiling temperature for 9 min, standing for 7 min and then followed by another sub-boiling temperature for 7 min. Sections were washed three times for 5 min each in PBS (pH 7.4). 3% bovine serum albumin was added to block non-specific binding for 30 min. Sections were incubated with the P-tau (Thr212/Ser214) primary antibody (1:200, MN1060; Thermo Fisher Scientific, Waltham, MA, USA), Aβ primary antibody (1:200, GB11197; Wuhan Servicebio Technology Co., Ltd.), or P-tau (Thr205/Ser202) primary antibody (1:200, GB113883; Wuhan Servicebio Technology Co., Ltd.) overnight at 4°C, washed three times for 5 min each in PBS, and incubated with secondary antibodies (HRP labeled) goat anti-rabbit (1:200, GB23303; Wuhan Servicebio Technology Co., Ltd.) or goat anti-mouse (1: 200, GB23301; Wuhan Servicebio Technology Co., Ltd.) at room temperature for 50 min. After three 5-min washed in PBS, DAB chromogenic agent (G1211; Wuhan Servicebio Technology Co., Ltd.) was added. After nucleus counterstaining and dehydration through graded alcohols, sections were sealed and examined with a microscope.

### 2.12. Statistical methods

Data were statistically analyzed using SPSS 22.0 software (IBM Corp., Armonk, NY, USA). Quantitative data were normalized and are expressed as mean ± SEM. Comparison between groups were performed using one-way ANOVA, and Least significance difference (LSD) *post hoc* test. The MWM latency data were analyzed using a repeated-measures analysis of variance. All tests were two-sided with an α of 0.05 and statistical significance threshold of *P* < 0.05.

## 3. Results

### 3.1. Bioactive components of YQF

**FIGURE 1:**
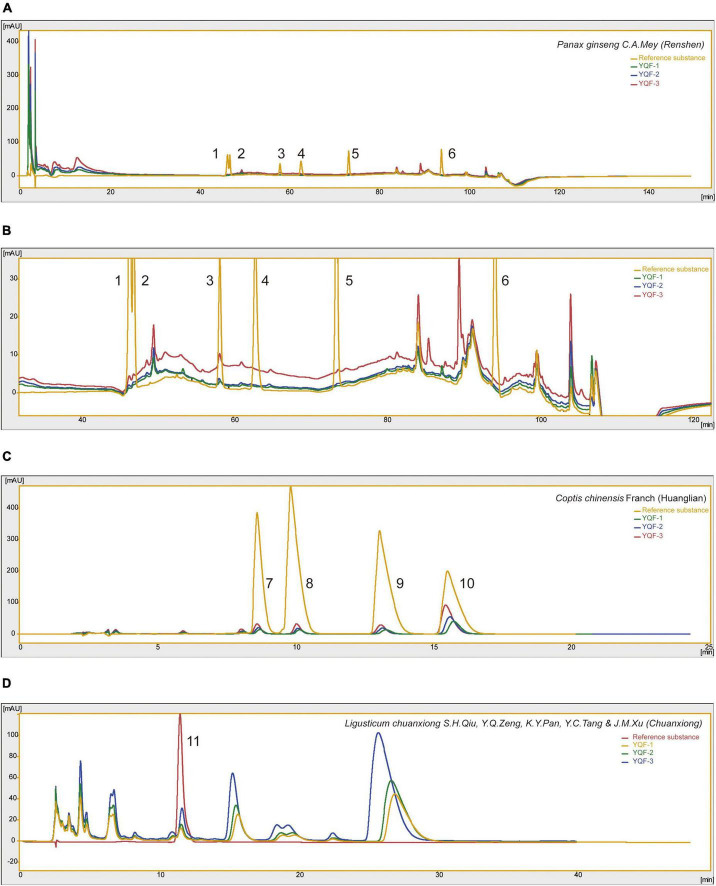
The chromatographic profile of Yizhiqingxin formula (YQF). Major compounds of **(A,B)** *Panax ginseng* C. A. Mey (Renshen), **(C)** *Coptis chinensis* Franch (Huanglian) and **(D)** *Ligusticum chuanxiong* S. H. Qiu, Y. Q. Zeng, K. Y. Pan, Y. C. Tang, and J. M. Xu (Chuanxiong) in YQF compared to reference standards, 1: ginsenosides Rg1; 2: ginsenosides Re; 3: ginsenosides Rb1; 4: ginsenosides Rg2; 5: ginsenosides Rd; 6: ginsenosides Rh2; 7: epiberberine; 8: coptisine chloride; 9: palmatine; 10: berberine; 11: ferulic acid.

Yizhiqingxin formula (YQF) consist of *Panax ginseng* C.A.Mey (Renshen), *Coptis chinensis* Franch (Huanglian), and *Ligusticum chuanxiong* S.H.Qiu, Y.Q.Zeng, K.Y.Pan, Y.C.Tang and J.M.Xu (Chuanxiong) in a ratio of 9:5:6. High-Performance Liquid Chromatography (HPLC) Analysis of YQF showed the main bioactive components in Figure 1. Specifically speaking, ginsenosides Rg1, ginsenosides Re, ginsenosides Rd, ginsenosides Rh2 in *Panax ginseng* C.A.Mey (Renshen) (Figures 1A, B); epiberberine, coptisine chloride, palmatine, and berberine in *Coptis chinensis* Franch (Huanglian) (Figure 1C); ferulic acid in *Ligusticum chuanxiong* S.H.Qiu, Y.Q.Zeng, K.Y.Pan, Y.C.Tang and J.M.Xu (Chuanxiong) (Figure 1D). Table 1 shows the quantification of bioactive components of YQF. The contents of each component were the most in YQF-3 and the least in YQF-1.

**TABLE 1:** Quantification of bioactive components of Yizhiqingxin formula (YQF).

Reference standards	YQF-1 (mg/g)	YQF-2 (mg/g)	YQF-3 (mg/g)
ginsenosides Rg1	10.25	17.33	47.74
ginsenosides Re	–	14.10	38.16
ginsenosides Rb1	11.37	16.81	71.31
ginsenosides Rg2	–	–	–
ginsenosides Rd	–	–	58.92
ginsenosides Rh2	–	–	–
epiberberine	25.99	26.77	51.76
coptisine chloride	23.64	27.87	60.60
palmatine	25.57	35.81	65.74
berberine	115.35	157.08	326.68
ferulic acid	203.92	288.43	826.92

There is a good linear relationship between the concentration of the reference standards and the Peak area (mAU.s), and external standard method was used for quantitative analysis.

### 3.2. Effects of YQF on learning and memory ability

**FIGURE 2:**
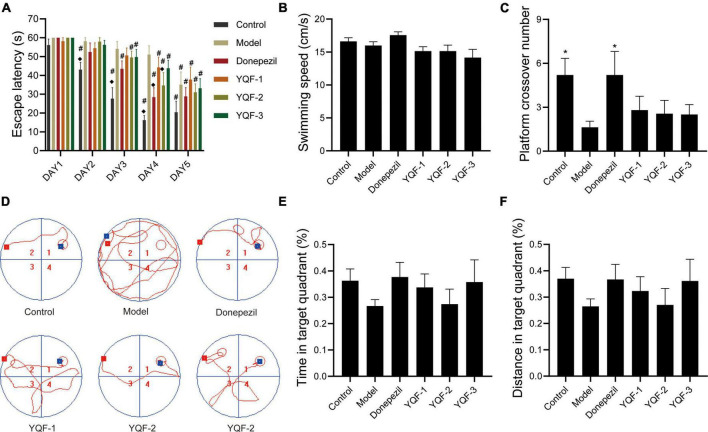
Effects on learning and memory ability in Alzheimer’s disease (AD) mice; Latency data were analyzed by ANOVA with repeated measures design information, and other data were analyzed by one-way ANOVA, presented as mean ± SEM, *n* = 8–10; *P < 0.05, compared to the model group. **(A)** Escape latency, **(B)** swimming speed, **(C)** platform crossover number, **(D)** representative swimming paths, **(E)** time in target quadrant, and **(F)** distance in target quadrant. ^◆^*P* < 0.05 for latency data between groups on the same day compared with the model group, and ^#^*P* < 0.05 for latency data within groups compared with the first day.

There were no significant differences in the swimming speeds of within in each group (*P* > 0.05), and effects on locomotor ability could be excluded. Compared to the control group, the model group showed significantly longer escape latencies (*P* = 0.000) and significantly fewer platform crossings (*P* = 0.026), suggesting impaired spatial learning memory ability in AD mice. The following intra-group comparisons were performed: after 5 days of repeated training (twice daily), compared to day 1, escape latency was significantly shorter starting on day 3 in the Donepezil (*P* = 0.003), YQF-2 (*P* = 0.014), and YQF-3 (*P* = 0.039) groups, and day 4 in the YQF-1 group (*P* = 0.032). Escape latency was reduced to different degrees on the training days, suggesting that experimental mice formed a stable spatial reference memory for the fixed platform position. Between-group comparisons showed that the escape latency in the donepezil group was significantly lower on day 4 than that of the model group (*P* = 0.003); on the same day, compared to the model group, escape latencies in YQF-2 group were significantly lower (*P* = 0.029). These results suggested that donepezil and YQF-2 could improve the learning and memory abilities of AD mice. These results are depicted in Figure 2 and Supplementary data 1.

### 3.3. Histopathological changes of the hippocampus

**FIGURE 3:**
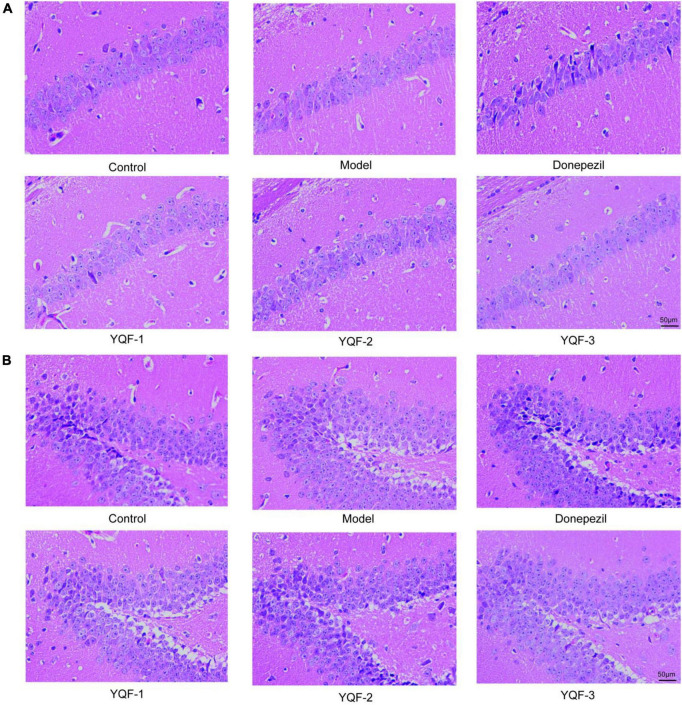
Pathological morphology of the hippocampus in each group; Hematoxylin–eosin (HE) staining of the CA1 region **(A)** of the hippocampus and dentate gyrus (DG) region **(B)** in each group, *n* = 3.

HE staining was used to observe changes in hippocampal morphology in each group (Figure 3). Compared to the control group, more intensely stained eosinophilic cells were observed in the model group, with a relatively loose cell arrangement, reduced cell number, and unclear or absent nuclei. The donepezil and YQF groups showed different degrees of improvement, with clear cell borders, relatively neat and dense arrangement, clear nucleoli, fewer eosinophilic cells, and more cells with blue-purple cytoplasm, suggesting that the administration of donepezil could reduce neuronal signs of aging and neurodegeneration and repair pathological signs in the hippocampus. The morphologies of the donepezil and YQF-2 groups were clearly different from the other groups.

### 3.4. Effects of YQF on hippocampal neurons

**FIGURE 4:**
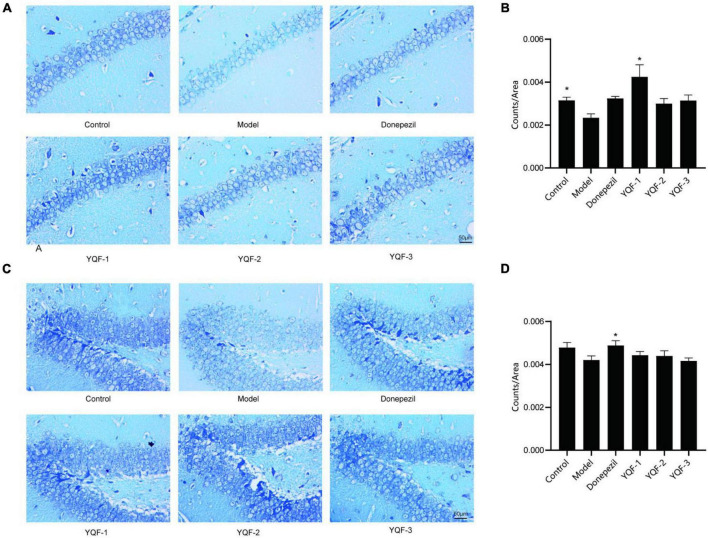
Effects on hippocampal neurons in each group; Nissl staining of the CA1 **(A,B)** and dentate gyrus (DG) regions **(C,D)** of the hippocampus in each group. Data were analyzed by one-way ANOVA, presented as mean ± SEM, *n* = 3, *P < 0.05 compared to the model group.

The effect of YQF on neurons was evaluated using Nissl staining (Figure 4). Morphologically, neurons in the CA1 and dentate gyrus (DG) regions of the hippocampus in the control group were neatly and densely organized, with deep cytoplasmic staining and a high number of Nissl bodies. Neurons in the model group showed relatively lighter blue staining, with a reduced number of Nissl bodies and relatively loosely arranged cells. In the YQF groups, there was stronger cytoplasmic staining and neurons were densely arranged. Quantitatively, in the CA1 region of the hippocampus, neuron numbers were significantly higher in the YQF-1 (*P* = 0.000) group compared to the model group, while neuron numbers were significantly higher in the donepezil group (*P* = 0.026) in the DG region of the hippocampus, suggesting that YQF can protect hippocampal neurons to some extent and prevent degenerative necrosis.

### 3.5. Effects of YQF on Aβ pathology in the hippocampus

**FIGURE 5:**
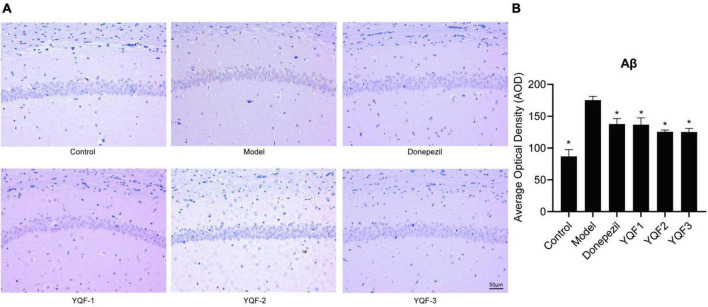
Effects on Aβ pathology in the hippocampus in each group; Aβ pathology of the hippocampus CA1 region **(A)** and quantitative analysis **(B)** in each group. Data were analyzed by one-way ANOVA, presented as mean ± SEM, *n* = 3, *P < 0.05 compared to the model group.

Immunohistochemistry was used to evaluate the effects of YQF on Aβ pathology in the CA1 region of the hippocampus. Compared to the control group, there was significantly more Aβ labeling in the CA1 region in model mice, and positive staining in the CA1 region was significantly lower in the donepezil (*P* = 0.006), YQF-1 (*P* = 0.005), YQF-2 (*P* = 0.001), and YQF-3 (*P* = 0.001) groups (Figure 5).

### 3.6. Effects of YQF on tau protein hyperphosphorylation in the hippocampus

**FIGURE 6:**
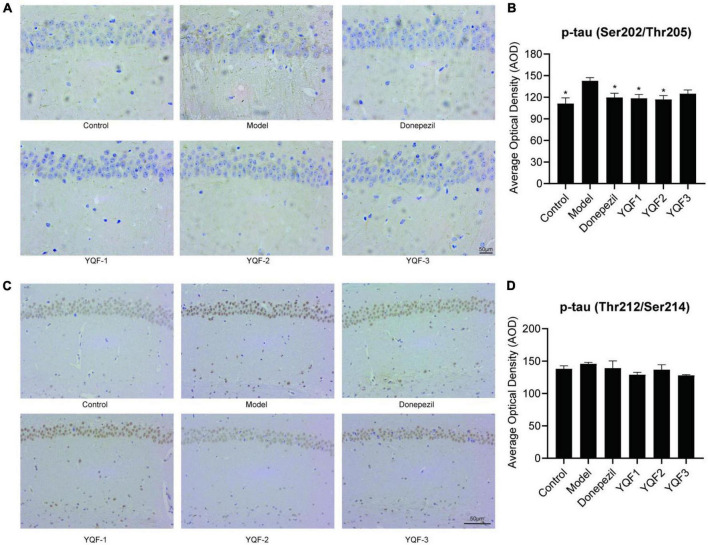
Effects on tau protein hyperphosphorylation in the hippocampus in each group; Immunohistochemically labeled p-tau (Ser202/Thr205) **(A,B)** and p-tau (Thr212/Ser214) **(C,D)** in each group. Data were analyzed by one-way ANOVA, presented as mean ± SEM, *n* = 3; *P < 0.05, compared to the model group.

Immunohistochemistry was used to evaluate the effects of YQF on tau hyperphosphorylation in the CA1 region of the hippocampus. Compared to the control group, p-tau labeling (Ser202/Thr205) was significantly higher in the model group and lower in the donepezil (*P* = 0.012), YQF-1 (*P* = 0.009), and YQF-2 (*P* = 0.006) groups (Figures 6A, B). And p-tau (Ser214/Thr212) labeling has the same tendency (Figures 6 C, D).

### 3.7. Effects of YQF on serum inflammatory factors and chemokines

**FIGURE 7:**
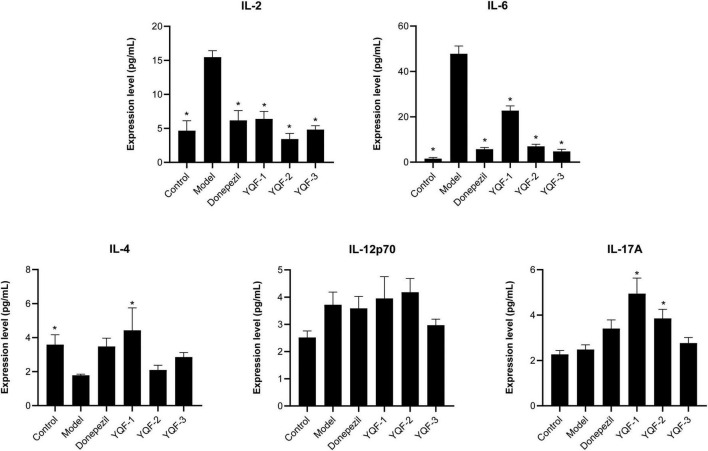
Effects on serum inflammatory factors in each group; data were analyzed by one-way ANOVA, presented as mean ± SEM, *n* = 6; *P < 0.05, compared to the model group.

Serum inflammatory factors and chemokines were evaluated using a high-throughput serum multifactor assay. Regarding the serum inflammatory factors (Figure 7), compared to the control group, serum interleukin IL-2 (*P* = 0.000), IL-6 (*P* = 0.000) were significantly higher in the model group and IL-4 was significantly lower in the model group (*P* = 0.043). The donepezil, YQF-1, YQF-2, and YQF-3 groups showed significantly lower expression of IL-2 and IL-6 (*P* = 0.000), and with significantly higher IL-4 levels in the YQF-1 group (*P* = 0.007).

**FIGURE 8:**
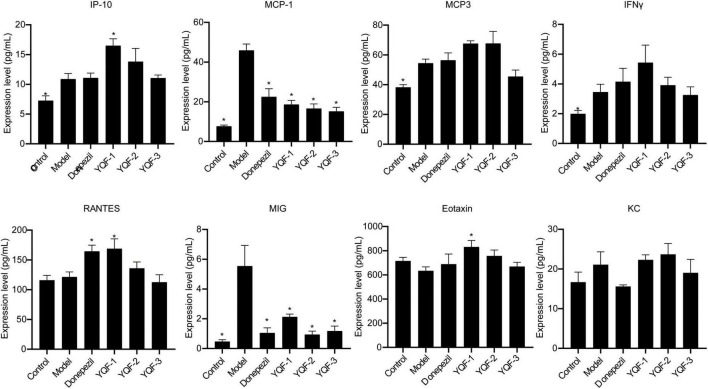
Effects on serum chemokines in each group; data were analyzed by one-way ANOVA, presented as mean ± SEM, *n* = 6; *P < 0.05, compared to the model group.

Regarding the serum chemokines (Figure 8), compared to the control group, Interferon-gamma induced protein 10 (IP-10) (*P* = 0.042), monocyte chemotactic protein (MCP)-1 (*P* = 0.000), monocyte chemotactic protein (MCP)-3 (*P* = 0.022), Interferon gamma (INFγ) (*P* = 0.034), and monokine induced by IFN-γ (MIG) (*P* = 0.000) were significantly higher in the model group, while regulated activation normal T cell expressed and secreted (RANTES), exotaxin, and KC were not significantly different between the control and model groups. The donepezil, YQF-1, YQF-2, and YQF-3 groups showed significantly reduced MCP-1 and MIG levels (*P* = 0.000), suggesting reduced peripheral inflammation levels. Additionally, the YQF-1 group showed significantly higher RANTES (*P* = 0.010), γ-inducible protein (IP)-10 (*P* = 0.003) and Eotaxin levels (*P* = 0.005).

## 4. Discussion

Patients with AD generally have a long illness duration and poor compliance; therefore, compared to the traditional individual Chinese medicine compound decoction method, the preparations of Chinese medicines based on the established modern Chinese medicine compound extraction processes are stable and convenient to take, which makes them more suitable for “simple, effective, and inexpensive” medicine and long-term use by patients with AD. The development of a Chinese medicine compound with definite efficacy in AD treatment will be beneficial, showing the therapeutic advantages of TCM and meeting the demand for new drug developments. Previous studies have confirmed that crude extract of YQF can effectively improve spatial learning and memory abilities of aged rats and APP/PS1 model mice, improve histopathological and morphological characteristics in rats, significantly increase acetylcholine and IL-10 levels, reduce the expression of inflammatory factors such as TNF-α and IL-6, and activate the BDNF/TrkB pathway to exert neuroprotective effects, suggesting that it has good clinical prospects in the treatment of AD (Yang et al., 2019; Ma et al., 2020).

The water extraction method is close to the “traditional preparation,” with low cost and a simple process, but the scope of its application is small, and many active ingredients with small polarity are not easily extracted, water-soluble components such as polysaccharides, glycosides and alkaloids can be easily extracted. Alcohol precipitation method is a purification and refining method commonly used in water extraction of TCM. By alternating treatment of water and different concentrations of ethanol, alkaloids, glycosides, amino acids, organic acids and other components can be retained, and impurities such as protein, gelatinized starch, lipid-soluble pigment, resin, and some sugars can be removed to further enrich the effective components. Ensure the maximum utilization of raw materials. Ethanol has better solubility, shorter extraction time, and less dissolved water-soluble impurities. Ethanol is a common organic solvent that can increase the extraction rate and is less toxic but flammable. The main active components in *Panax ginseng* C.A.Mey (Renshen) are ginsenosides, and ethanol extraction can reduce the dissolution of other impurities. The main components of *Coptis chinensis* Franch (Huanglian) are alkaloids and flavonoids, which have certain solubility in water. For *Ligusticum chuanxiong* S.H.Qiu, Y.Q.Zeng, K.Y.Pan, Y.C.Tang and J.M.Xu (Chuanxiong), the following two treatment methods can be used: (1) Extracting Chuanxiong with other herbs by decocting; (2) The volatile oil of Chuanxiong was extracted by steam distillation, and the residue of Chuanxiong was extracted by decocting together with other herbs. Considering that there are few kinds of materials containing volatile oil in YQF, and the content of volatile oil in Chuangxiong is low, in order to simplify the technological process, it is proposed to use the decoction method to extract Chuangxiong together with other herbs. In order to complete the preclinical study of the innovative TCM YQF for the treatment of AD, considering the pharmacological effects of each herb in YQF and the physical and chemical properties of the main components, we designed three preparations, namely “water extraction” (YQF-1), “water extraction and alcohol precipitation method” (YQF-2), and “alcohol extraction” (YQF-3). Specific extraction methods were determined by the Preparation Department of Xiyuan Hospital, Chinese Academy of Traditional Chinese Medicine.

The effective components of the three methods were preliminarily explored and quantitatively analyzed by HPLC (Figure 1 and Table 1). The main components in YQF are ginsenoside Re, ginsenoside Rg1, ginsenoside Rb1, epiberberine, coptisine chloride, palmatine, berberine, and ferulic acid. Different extraction processes have a great impact on the content of the effective components of the extracted drugs. The results showed that YQF-3 (alcohol extraction) has the highest content of active compounds, followed by YQF-2 (water extraction and alcohol precipitation method). The YQF-1 (water extraction) has the lowest content of active compounds. The active components of ginseng, protopanaxatriol saponins Re and Rg1, are more polar, while the protopanaxadiol saponin Rb1 contains more glycosyl groups (hydroxyl groups), making aqueous extraction more efficient. Alcohol extraction can increase the extraction rate of ginsenosides to diol-type saponins (Gong et al., 2014). The alkaloids berberine, epiberberine, xanthophylline, and palmatine, which are the main pharmacologically active components of *Coptis chinensis* Franch (Huanglian), are more polar and can be extracted with water or ethanol, but the extraction efficiency is higher in ethanol (Liu et al., 2006). Ferulic acid, a phenolic acid, has a high content in *Ligusticum chuanxiong* S.H.Qiu, Y.Q.Zeng, K.Y.Pan, Y.C.Tang and J.M.Xu (Chuanxiong), its water solubility is poor, but its alcohol solubility is good, and the ethanol extraction rate is relatively high (Yang et al., 2012). Differences in polarity, solubility, and other characteristics of the active ingredients of Chinese herbal medicines have led to slight differences in the compositions and contents of extracts in different solvents, which may be one of the reasons for the differences in the present results.

Ginsenoside Rg1, ginsenoside Re, and coptisine have been detected in the brain of in SAMP8 mice after YQF treatment, showing protective roles (Yang Y. et al., 2017). Impaired learning memory capacity is one of the most significant clinical symptoms of AD, and pathological changes such as Aβ pathology, tau hyperphosphorylation, and inflammation-related indicators can reflect the severity of the disease and are often used as indicators of treatment efficacy. Studies have confirmed that a variety of active ingredients such as ginsenosides, alkaloids, and ferulic acid can play important roles in the prevention and treatment of AD by improving learning and memory and reducing AD pathology and inflammation levels *via* multiple pathways (Sheng et al., 2015; Yang W. T. et al., 2017; Jung et al., 2009; Singh et al., 2021). Ginsenosides negatively regulate β-secretase activity, where Rb1 and Rb2 exhibit a high binding affinity for β-secretase (Choi et al., 2016), while ginsenoside Re activates PPARγ (Cao et al., 2016), ginsenoside Rg1 suppresses PPARγ-regulated BACE1 activity (Chen et al., 2012), and ginsenoside Rd increases sAPPα expression levels (Yan et al., 2017), which ultimately reduces neurotoxic Aβ production. In addition, ginsenoside Rb1 and Rd can inhibit tau protein hyperphosphorylation (Li et al., 2011; Zhao et al., 2013). Berberine reduces Aβ in the brain of AD mice (Ye et al., 2021; Wang Y. Y. et al., 2021), and reduces the tau hyperphosphorylation in the hippocampus of AD mice (He et al., 2017). Ferulic acid also improved Aβ plaque deposition and spatial memory deficits (Wang N. Y. et al., 2021).

It is of great significance for developing safe, effective, quality-controlled and innovative Chinese medicines to screen and evaluate the rationality of the extraction process by pharmacodynamic indicators. To further explore these preparation processes and their efficacy and provide an experimental basis for future translational studies, the present study evaluated three preparations, namely “water extraction” (YQF-1), “water extraction and alcohol precipitation method” (YQF-2), and “alcohol extraction” (YQF-3), in AD treatment. The 3 × Tg AD mice selected for this study are an ideal AD model showing obvious behavioral abnormalities after 6 months of age and age-related Aβ pathology and tau hyperphosphorylation (Oddo et al., 2003; Billings et al., 2005). In the present study, 7–8-month-old AD mice were evaluated for the efficacy of three YQF extraction processes after 2 months of treatment, showing the following results: (1) different degrees of improved spatial learning and memory and histopathological morphology in AD mice, with more significant effects for YQF-2; (2) different degrees of protective effects on hippocampal neuron morphology, with the most significant effects on CA1 neuron number for YQF-1; (3) the effects of YQF-1, YQF-2 and YQF-3 on Aβ pathology in the CA1 region were both significant (4) reduced hyperphosphorylation of tau Ser202/Thr205 sites, with no effect of YQF-3; (5) reduced expression of serum pro-inflammatory factors IL-2, IL-6 and serum chemokines MCP-1, MIG. Furthermore, YQF-1 not only significantly increased the level of anti-inflammatory factor IL-4 but also increased the expression of pro-inflammatory factor IL-17A. RANTES, IP-10 and Eotaxin were also increased after YQF-1 intervention, but the roles of RANTES, IP-10 and Eotaxin remain controversial in AD.

The present study confirmed the efficacy of YQF as an adjunctive treatment in AD model mice. In general, although the extract contents of the three processes are slightly different, YQF of the three processes can significantly improve the main pathological features of AD, Aβ and hyperphosphorylation of tau protein, and can reduce the level of inflammation in serum. Besides, YQF-2 can significantly improve the learning and memory ability, while YQF-1 and YQF-3 have no significant effect; YQF-1 had a statistically significant protective effect on neurons, and YQF-2 and YQF-3 had the same trend. Limited by the experimental conditions, we only made a rough analysis and quantification of some possible components of YQF. However, the chemical composition of Chinese herbal compounds is complex, there are still many effective components that could not be analyzed in this experiment. Moreover, the number of experimental animals in each group is relatively small, which may also lead to the differences in the above results. In the follow-up studies, more systematic medicinal chemistry and pharmacokinetics studies are needed to optimize the extraction process considering economic costs and the feasibility of industrialization and promotion in addition to comparisons of pharmacodynamics to screen for materials. Different experimental models and efficacy indicators, and expand sample size are also needed to further provide a basis for preclinical research of YQF.

## Data availability statement

The raw data supporting the conclusions of this article will be made available by the authors, without undue reservation.

## Ethics statement

The animal study was reviewed and approved by the Ethics Committee of the Xiyuan Hospital of the China Academy of Chinese Medical Sciences (No. 2021XLC032-2).

## Author contributions

WW performed the experiments and data analysis and wrote the manuscript. RZ, Q-YH, and S-RC assisted in the experiments. HP and L-NM assisted with the data analysis. YC and HL conceived the study, designed the experiments, and assisted in the modification of the manuscript. All authors reviewed and approved the manuscript.

## References

[B1] Billings L. M. Oddo S. Green K. N. McGaugh J. L. LaFerla F. M. (2005). Intraneuronal Abeta causes the onset of early Alzheimer’s disease-related cognitive deficits in transgenic mice. *Neuron* 45 675–688. 10.1016/j.neuron.2005.01.040 15748844

[B2] Cao G. Su P. Zhang S. Guo L. Zhang H. Liang Y. (2016). Ginsenoside Re reduces Aβ production by activating PPARγ to inhibit BACE1 in N2a/APP695 cells. *Eur. J. Pharmacol.* 793 101–108. 10.1016/j.ejphar.2016.11.006 27840193

[B3] Chen L. M. Lin Z. Y. Zhu Y. G. Lin N. Zhang J. Pan X. D. (2012). Ginsenoside Rg1 attenuates β-amyloid generation via suppressing PPARγ-regulated BACE1 activity in N2a-APP695 cells. *Eur. J. Pharmacol.* 675 15–21. 10.1016/j.ejphar.2011.11.039 22166376

[B4] Choi R. J. Roy A. Jung H. J. Ali M. Y. Min B. S. Park C. H. (2016). BACE1 molecular docking and anti-Alzheimer’s disease activities of ginsenosides. *J. Ethnopharmacol.* 190 219–230. 10.1016/j.jep.2016.06.013 27275774

[B5] Gong X. Zhang Y. Pan J. Qu H. (2014). Optimization of the ethanol recycling reflux extraction process for saponins using a design space approach. *PLoS One* 9:e114300. 10.1371/journal.pone.0114300 25470598 PMC4255001

[B6] Guo C. (2014). Simultaneous determination of sixteen ginsenosides in Panax ginseng and its preparation by HPLC. *Chin. Traditional Herb. Drugs* 45 2009–2013.

[B7] He W. Wang C. Chen Y. He Y. Cai Z. (2017). Berberine attenuates cognitive impairment and ameliorates tau hyperphosphorylation by limiting the self-perpetuating pathogenic cycle between NF-κB signaling, oxidative stress and neuroinflammation. *Pharmacol. Rep.* 69 1341–1348. 10.1016/j.pharep.2017.06.006 29132092

[B8] Jung H. A. Min B. S. Yokozawa T. Lee J. H. Kim Y. S. Choi J. S. (2009). Anti-Alzheimer and antioxidant activities of Coptidis Rhizoma alkaloids. *Biol. Pharm. Bull.* 32 1433–1438. 19652386 10.1248/bpb.32.1433

[B9] Li L. Liu J. Yan X. Qin K. Shi M. Lin T. (2011). Protective effects of ginsenoside Rd against okadaic acid-induced neurotoxicity in vivo and in vitro. *J. Ethnopharmacol.* 138 135–141. 10.1016/j.jep.2011.08.068 21945003

[B10] Liu B. Li W. Chang Y. Dong W. Ni L. (2006). Extraction of berberine from rhizome of Coptis chinensis Franch using supercritical fluid extraction. *J. Pharm. Biomed. Anal.* 41 1056–1060. 10.1016/j.jpba.2006.01.034 16500064

[B11] Ma L. Cao Y. Wang F. Li Z. Wang Z. Yang Y. (2020). Yizhi qingxin formula extract ameliorates cognitive decline in aged rats via the brain-derived neurotrophic Factor/Tropomyosin receptor Kinase B pathway. *Front. Pharmacol.* 11:510. 10.3389/fphar.2020.00510 32425777 PMC7203446

[B12] Oddo S. Caccamo A. Shepherd J. D. Murphy M. P. Golde T. E. Kayed R. (2003). Triple-transgenic model of Alzheimer’s disease with plaques and tangles: intracellular Abeta and synaptic dysfunction. *Neuron* 39 409–421. 10.1016/S0896-6273(03)00434-3 12895417

[B13] Pei H. Ma L. Cao Y. Wang F. Li Z. Liu N. (2020). Traditional Chinese Medicine for Alzheimer’s Disease and Other Cognitive Impairment: A Review. *Am. J. Chin. Med.* 48 487–511. 32329645 10.1142/S0192415X20500251

[B14] Scheltens P. De Strooper B. Kivipelto M. Holstege H. Chételat G. Teunissen C. E. (2021). Alzheimer’s disease. *Lancet* 397 1577–1590. 33667416 10.1016/S0140-6736(20)32205-4 PMC8354300

[B15] Sheng C. Peng W. Xia Z. A. Wang Y. Chen Z. Su N. (2015). The impact of ginsenosides on cognitive deficits in experimental animal studies of Alzheimer’s disease: a systematic review. *BMC Compl. Altern. Med.* 15:386. 10.1186/s12906-015-0894-y 26497388 PMC4619356

[B16] Singh Y. P. Rai H. Singh G. Singh G. K. Mishra S. Kumar S. (2021). A review on ferulic acid and analogs based scaffolds for the management of Alzheimer’s disease. *Eur. J. Med. Chem.* 215 113278. 10.1016/j.ejmech.2021.113278 33662757

[B17] Wang N. Y. Li J. N. Liu W. L. Huang Q. Li W. X. Tan Y. H. (2021). Ferulic Acid Ameliorates Alzheimer’s Disease-like Pathology and Repairs Cognitive Decline by Preventing Capillary Hypofunction in APP/PS1 Mice. *Neurotherapeutics* 18 1064–1080. 10.1007/s13311-021-01024-7 33786807 PMC8423929

[B18] Wang Y. Y. Yan Q. Huang Z. T. Zou Q. Li J. Yuan M. H. (2021). Ameliorating Ribosylation-Induced Amyloid-β Pathology by Berberine via Inhibiting mTOR/p70S6K Signaling. *J. Alzheimers Dis.* 79 833–844. 10.3233/JAD-200995 33361598

[B19] Yan X. Hu G. Yan W. Chen T. Yang F. Zhang X. (2017). Ginsenoside Rd promotes non-amyloidogenic pathway of amyloid precursor protein processing by regulating phosphorylation of estrogen receptor alpha. *Life Sci.* 168 16–23. 10.1016/j.lfs.2016.11.002 27825720

[B20] Yang W. T. Zheng X. W. Chen S. Shan C. S. Xu Q. Q. Zhu J. Z. (2017). Chinese herbal medicine for Alzheimer’s disease: Clinical evidence and possible mechanism of neurogenesis. *Biochem. Pharmacol.* 141 143–155. 28690138 10.1016/j.bcp.2017.07.002

[B21] Yang X. Wu X. Hu L. Guo H. (2012). [Effect of different solvents on extraction of effective components from Ligusticum chuanxiong]. *Zhongguo Zhong Yao Za Zhi* 37 1942–1945. 23019875

[B22] Yang Y. Jia X. Feng J. Wang Z. Cao Y. Liu J. (2017). Fuzheng quxie decoction ameliorates learning and memory impairment in SAMP8 mice by decreasing tau hyperphosphorylation. *Evid. Based Complement. Alternat. Med.* 2017 5934254. 10.1155/2017/5934254 29422936 PMC5750500

[B23] Yang Y. Wang Z. Cao Y. Liu J. Li P. Li H. (2019). Yizhiqingxin formula alleviates cognitive deficits and enhances autophagy via mtor signaling pathway modulation in early onset Alzheimer’s disease mice. *Front. Pharmacol.* 10:1041. 10.3389/fphar.2019.01041 31607908 PMC6758600

[B24] Ye C. Liang Y. Chen Y. Xiong Y. She Y. Zhong X. (2021). Berberine improves cognitive impairment by simultaneously impacting cerebral blood flow and β-amyloid accumulation in an APP/tau/PS1 mouse model of Alzheimer’s disease. *Cells* 10:1161. 10.3390/cells10051161 34064687 PMC8150323

[B25] Zhao H. H. Di J. Liu W. S. Liu H. L. Lai H. Lü Y. L. (2013). Involvement of GSK3 and PP2A in ginsenoside Rb1’s attenuation of aluminum-induced tau hyperphosphorylation. *Behav. Brain Res.* 241 228–234. 10.1016/j.bbr.2012.11.037 23219964

