# Supplementary material for: Comparison of Yizhiqingxin formula extraction methods and their pharmacodynamic differences
Source: Front Neurosci. 2023 Feb 16;17:1097859. doi: 10.3389/fnins.2023.1097859 (PMC9978475; doi:10.3389/fnins.2023.1097859)
Supplement: Supplementary file 1 [file Table_1.docx]

Table 1 Latency results of experimental positioning navigation for each group of mice

| Groups | Positioning navigation experiment | | | | |
| --- | --- | --- | --- | --- | --- |
|  | DAY1 | DAY2 | DAY3 | DAY4 | DAY5 |
| Control^*^ | 56.10±1.58 | 43.08±3.16^◆#^ | 27.63±4.21^◆#^ | 16.23±4.74^◆#^ | 20.42±5.42^#^ |
| Model | 60.00±1.77 | 58.12±3.54 | 54.06±4.71 | 51.06±5.30 | 35.08±6.06^#^ |
| Donepezil ^*^ | 60.00±1.58 | 52.44±3.16 | 43.50±4.21^#^ | 28.41±4.74^◆#^ | 28.87±5.42^#^ |
| YQF- 1 | 58.17±1.58 | 54.47±3.16 | 50.58±4.21 | 44.26±4.74^#^ | 37.76±5.42^#^ |
| YQF- 2 | 60.00±1.67 | 57.96±3.33 | 49.55±4.44^#^ | 34.64±5.00^◆#^ | 31.06±5.71^#^ |
| YQF- 3 | 60.00±1.77 | 56.21±3.54 | 49.77±4.71^#^ | 43.76±5.30^#^ | 33.18±6.06^#^ |

Note: Latency data were presented as Mean with SEM using ANOVA with repeated measurement data, with ^*^*P*<0.05 for 5-day latency data between groups compared with the model group; ^◆^*P*<0.05 for latency data between groups on the same day compared with the model group; and ^#^*P*<0.05 for latency data within groups compared with the first day.

Table 2 Results of spatial exploration experiments for each group of mice

| Groups | Space exploratory experiments | | |
| --- | --- | --- | --- |
|  | Platform crossover number | Distance in target quadrant (%) | Time in target quadrant (%) |
| Control | 5.20±1.14^*^ | 0.37±0.04 | 0.36±0.05 |
| Model | 1.63±0.42 | 0.27±0.03 | 0.27±0.02 |
| Donepezil | 5.20±1.61^*^ | 0.37±0.06 | 0.38±0.06 |
| YQF-1 | 2.80±0.95 | 0.32±0.05 | 0.34±0.05 |
| YQF-2 | 2.56±0.92 | 0.27±0.06 | 0.27±0.06 |
| YQF-3 | 2.50±0.68 | 0.36±0.08 | 0.36±0.08 |

Note: Data are presented as Mean with SEM using one-way Analysis of Variance ANOVA, with **P*<0.05 compared to the model group.
